# Diminished Thrombogenic Responses by Deletion of the *Podocalyxin Gene* in Mouse Megakaryocytes

**DOI:** 10.1371/journal.pone.0026025

**Published:** 2011-10-07

**Authors:** Miguel Pericacho, Sonia Alonso-Martín, Susana Larrucea, Consuelo González-Manchón, Darío Fernández, Inés Sánchez, Matilde S. Ayuso, Roberto Parrilla

**Affiliations:** 1 Department of Cellular and Molecular Medicine, Centro de Investigaciones Biológicas (CSIC), Madrid, Spain; 2 CIBER de Enfermedades Raras (CIBERER), Madrid, Spain; 3 Laboratorio de Inmunología, Hospital de Cruces, Baracaldo, Spain; Heart Center Munich, Germany

## Abstract

Podocalyxin (Podxl) is a type I membrane sialoprotein of the CD34 family, originally described in the epithelial glomerular cells of the kidney (podocytes) in which it plays an important function. Podxl can also be found in megakaryocytes and platelets among other extrarenal places. The surface exposure of Podxl upon platelet activation suggested it could play some physiological role. To elucidate the function of Podxl in platelets, we generated mice with restricted ablation of the *podxl* gene in megakaryocytes using the Cre-LoxP gene targeting methodology. Mice with Podxl-null megakaryocytes did not show any apparent phenotypical change and their rates of growth, life span and fertility did not differ from the floxed controls. However, Podxl-null mice showed prolonged bleeding time and decreased platelet aggregation in response to physiological agonists. The number, size-distribution and polyploidy of Podxl-null megakaryocytes were similar to the floxed controls. Podxl-null platelets showed normal content of surface receptors and normal activation by agonists. However, the mice bearing Podxl-null platelets showed a significant retardation in the ferric chloride-induced occlusion of the carotid artery. Moreover, acute thrombosis induced by the i.v. injection of sublethal doses of collagen and phenylephrine produced a smaller fall in the number of circulating platelets in Podxl-null mice than in control mice. In addition, perfusion of uncoagulated blood from Podxl-null mice in parallel flow chamber showed reduced adhesion of platelets and formation of aggregates under high shear stress. It is concluded that platelet Podxl is involved in the control of hemostasis acting as a platelet co-stimulator, likely due to its pro-adhesive properties.

## Introduction

Podocalyxin (Podxl), also known as Myb-Ets transformed progenitor (MEP), is a ∼160 KDa, highly sialylated and sulfated, membrane type I mucoprotein of the CD34 family [1]. This protein was first reported in the epithelial cells, podocytes, lining the luminal face of kidney glomeruli [2]. The podocytes are injured in many glomerular diseases, including minimal change disease (MCD), focal segmental glomerulosclerosis (FSGS), collapsing glomerulopathy, diabetic nephropathy, membranous glomerulopathy, crescentic glomerulonephritis, and lupus nephritis [3]–[5]. Those inflammatory processes are accompanied by loss of the normal architecture, “effacement”, of the luminal surface of podocytes and loss of the urinary filtration slits [3], [6]. Moreover, total podocalyxin knock out mice die of anuria within the first few hours after birth [7]. Podxl has also been found in vascular endothelium, lung, brain, multipotent hematopoietic precursors and platelets [8]–[10], and in several types of tumors in which the expression levels appear to correlate with the metastatic capacity [11]–[13]. Despite the consistent data indicating the important role played by Podxl in maintaining a normal renal function, almost nothing is known about its physiological role in extrarenal tissues.

Podxl is exposed on the plasma membrane of platelets upon stimulation by agonists [14] suggesting that it could cooperate in maintaining a normal hemostatic responsiveness. Moreover, the loss-of-function mutation (*plt1* mice) in the glycoprotein-*N*-acetylgalactosamine-3-galactosyltransferase (C1GalT1), essential for the synthesis of extended mucin-type *O*-glycans, produced underglycosylation of podocalyxin and glycoprotein Ib (GPIb) and caused thrombocytopenia and kidney disease, suggesting a contribution of the abnormally glycosylated Podxl to this pathology [15]. In agreement with these observations, we have recently reported a diminished adhesion of sialic acid deficient *O-*glycomutant CHO-Podxl cells compared with cells expressing normal sialylated Podxl [16]. These antecedents suggest that Podxl, like other mucin proteins, could play a role in cell adhesion/migration. In fact, a particular glycosylation pattern of Podxl could be responsible for the ability of high endothelial venules to bind L-selectin from other cells [17]–[19]. Moreover, CHO cells expressing recombinant human Podxl showed, P-selectin and integrin-dependent, enhanced adhesion and motility over immobilized ligands and increased cellular interactive capacity [16].

The present work aimed at investigating the functional role of Podxl in platelets. For this purpose, we used the Cre-LoxP gene targeting methodology to generate mice with specific ablation of the *podxl* gene in megakaryocytes. Mice with null-Podxl megakaryocytes showed prolonged bleeding time, decreased platelet aggregation in response to agonists, retarded ferric chloride-induced closure of the carotid artery, diminished *in vivo* thrombogenesis and decreased adherence of platelets under flow. These observations suggest that removal of the *podxl* gene from megakaryocytes perturbs the control of hemostasis.

## Methods

### Animals

Mice of the C57BL/6 strain, maintained under controlled conditions of light and temperature, were used in all the experiments. Podxl^lox/lox^:: Control or Podxl^lox/lox^:: Pf4-Cre littermates used in this study were generated on a mixed C57BL/6-129sv genetic background and were backcrossed for 10 generations with C57BL/6 mice. All animal experiments were done in such a way as to minimize the animal suffering, according to relevant national and international guidelines (“Guide for Care and Use of Laboratory at the Institute of Laboratory Animal Resources”).

The ethics committee of the Center for Biological Research (CSIC) and the grant review board (ANEP) of the Spanish Ministry of Science and Technology specifically approved this study in accordance with the guidelines of the European Community Council Directive 86/609 EEC.

### Generation of a conditional (floxed) podocalyxin allele

The DNA sequence of the podocalyxin gene was obtained by digestion of the murine genomic BACS 449o15 and 465p05 with *Bam*H I, *Eco*R I and *Xba* I. DNA fragments containing the *podxl* gene were identified by southern blot and cloned. Digestion of clone 465p05 with *Sac* I released a 4,381 bp fragment comprising exons 3 to 8 of *podxl* that was cloned into the *Sac* I site of pCR 2.1-TOPO.

**Figure 1:**
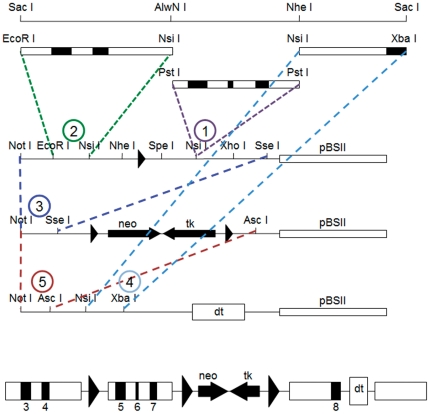
Podocalyxin gene targeting construct. Deletion of the *podxl gene*. Generation of the floxed *podxl* allele was performed as described in Methods. Circled numbers indicate the order of the different steps in the construction of the final chimera.

A conditional *podxl* transgene was built in three steps into the pBS II vector using the pLox plasmids [20], as shown in Fig. 1. The pLoxL contained the left homology arm (exons 3 and 4 of *podxl*); the pLoxC encompassed exons 5 to 7 of *podxl* flanked by LoxP sites and *neo* (PGK-NEO) and *tk* (HSV-TK) selection cassette; the pLoxR contained the right homology arm (exon 8 and part of the 3′-UTR of *podxl*) and a counterselection cassette for the diphteria toxin A chain (PGK-DT).

The left homology arm was generated by *Sac* I digestion of the genomic DNA fragment containing the *podxl* gene and amplification with oligonucleotide primers LoxL-S (5′-CCGAATTCCGCCTGCCTCAGCC-3′) to introduce an *Eco*R I site, and LoxL-AS (5′-GGATGCATTATCATGCCTCAGCTGG-3′) to replace the site *Alw*N I by a *Nsi* I site. The PCR product was cloned into AccepTor-Vector (AccepTor Vector Kits, Novagen). The pLoxL vector was modified by means of adapters to clone the PCR product into the *Eco*R I and *Nsi* I sites.

The genomic DNA fragment containing exon 8 and 3′-UTR of the *podxl* gene was amplified using oligonucleotides LoxR-S (5′-TCATGCATGCTTGTCTCTAACGCTG-3′) to replace a *Nsi* I site for *Nhe* I, and LoxR-AS (5′-GCTCTAGA GTGCAGTTGGGGTG-3′) that replaces a *Sac* I site for a *Xba* I. The PCR product was cloned in the AccepTor-Vector. We introduced an adapter into the pLoxR vector to generate *Nsi* I site for subcloning of the *podxl* right homology arm.

To generate the “floxed” fragment (exons 5 to 7 of *podxl*) we amplified a DNA fragment comprising exons 5 to 7 from the *Sac* I genomic DNA fragment using primers LoxC-S (5′-TACTGCAGCTGGGTCCAGAGCCTTA-3′) designed to replace the restriction site *Alw*N I by a *Pst* I site, and LoxC-AS (5′-AGCTGCAGTGACAGCGGGGACAAGA-3′) to replace an *Nhe* I site by *Pst* I site. The PCR product was cloned into AccepTor-Vector, released by digestion with *Pst* I, and cloned into the *Nsi* I site of the vector pLoxL. The pLoxC vector was digested with *Not* I and *Sse* I to clone the left homology arm and “floxed” fragments upstream of the *neo* and *tk* selection cassette. Finally, the *Not* I-*Asc* I fragment was subcloned upstream of the right homology arm in the pLoxR vector.

The final construction was sequence-verified using the following primers:

MPDL 939–957 (5′-GAGATGAGGTGTGTGAACT-3′)
MPDL 1053-1031 (5′-CTTATGGGAGGTTCACAGTT-3′)
MPDU 3 (5′-GACTAGCCATGCCTGAGTTTG -3′)
MPDU 1100 (5′-TGGTGGGGCTGCACTGCATA-3′)
MPDL 1272-1254 (5′-CGGCCTTAGGAGAAAGCTTCG3′)
MPDU 1211-1192 (5′-CTCTACATGTGGCCCCTATT-3′)
MPDU 1338-1318 (5′-CGGAGTCAGTGACATGAAGCT-3′)
MPDL i1484 (5′-ACTGTCTTAATGCTCTCATT-3′)
MPDL 1505-1486 (5′-CAGCTCCTCTGTGAGCCGTT-3′)
MPDU i7 (5′-TGAGATGGTCAGGCTAGTAGC-3′)

### Generation of mice with a floxed *podxl* gene

R1 E14 (129/Sv) embryonic stem (ES) cells, kindly donated by Dr. Nagy, were cultured according to their indications [21]. Twenty-five µg of the *podxl* construct were electroporated into ES cells. Positive selection was achieved with G418 (Sigma) at a concentration of 250 µg/mL and the resistant ES cell clones were screened for homologous recombination by PCR. The modified ES cells were expanded and transfected with the p-MC-Cre plasmid in order to remove the selection cassette (*neo* and *tk*). Finally, ES cells carrying the “floxed” *podxl* allele were used for morula aggregation to generate chimeric mice according to previous protocols [22]. Genotyping was carried out by PCR. Two founders were subsequently used for further analysis.

To generate mice with a restricted deletion of Podxl in platelets, the Podxl floxed mice were crossed to Pf4-Cre mice [23] (kindly donated by Dr. Skoda, Basel).

### Hemograms and tail bleeding time

Hemograms were determined in blood-EDTA samples withdrawn from the submaxillary vessels, using an automatic hemocytometer model Abacus Junior (Diatron, Diatron Messtechnik GmbH, Austria).

To determine the tail bleeding time the animals were anesthetized with isofluorane. The tail was transected at 5 mm from the tip and it was immediately immersed in 36°C phosphate buffered saline (PBS), and the time until cessation of bleeding recorded.

### Ferric chloride-induced closure of the carotid artery

The carotid artery blood flow was determine in mice anesthetized with isofluorane, while maintained the body temperature at 37°C on a heated blanket. The temperature was continuously recorded using a rectal miniprobe. The carotid artery was exposed and the blood flow measured with a 0.5 mm micro-flow probe (0.5 PBS) and the TS420 flow module (Transonic Systems, Inc., Ithaca, NY). The data was continuously recorded with a data acquisition system PowerLab 8/30, model ML870 (AD Instruments Ltd., Chalgrove, Oxfordshire OX44 7RW, UK).

To determine the carotid artery closure time, a 1 mm^2^ paper fragment soaked on 2% ferric chloride was placed for 2 minutes on the exposed carotid artery while the blood flow was continuously monitored. We considered the closure time to be that at which the blood-flow was interrupted for 3 minutes.

### Preparation and activation of platelets

Citrated blood samples were obtained by cardiac puncture under isofluorane anesthesia. The blood was centrifuged at 100 g for 5 minutes at room temperature. Platelet-rich plasma (PRP) was removed and centrifuged at 1300 g in the presence of 0.1 µg/mL prostacyclin and 0.02 U/mL apyrase (Sigma) for 5 minutes at room temperature, washed 3 times with modified Tyrode's buffer containing 0.1 µg/mL prostacyclin and 0.02 U/mL apyrase. The sedimented platelets were finally resuspended in Tyrode's buffer containing 0.02 U/mL apyrase and rested for 30 minutes at 37°C prior to stimulation.

The membrane content of surface receptors was determine in washed platelets, incubated with anti–GPIbα–DyLight649 antibody (Xia.G5), anti αIIbβ3-FITC (Leo.F2), FITC-labeled rat anti-mouse GPVI and FITC-labeled rat anti-mouse integrin alpha2 chain (CD49b) (Emfret Analytics, Eibelstadt, Germany) or control isotype-matched mouse IgGs for 30 minutes at room temperature. The samples were then washed and fixed with 1% formaldehyde, and analyzed in a cytometer EPICS XL FACS.

To determine the extent of platelets activation, 25 µL of washed platelets (10^6^ approximately) suspended in Tyrode's-buffer containing 1 mM CaCl_2_ were stimulated with ADP, thrombin or collagen. Activation was assessed by determining P-selectin exposure and αIIbβ3 activation by flow cytometry (cytometer model FC500) with a two-color analysis protocol using 5 µL of Dylight 649 anti-GPIbα moAb (Emfret Analytics) and 5 µL of FITC-anti-P-selectin (Beckton and Dickinson) or PE-anti-activated αIIbβ3 (JON/A, Emfret Analytics) moAbs. The extent of platelet activation was further assessed by measuring the surface exposure of phosphatidylserine, as indicated by the binding of FITC-Annexin V (Immunostep, Salamanca, Spain).

### Determination of prothrombin time (PT) and activated partial thromboplastin time (aPTT)

The concentration of PT and aPTT were determined in plasma samples in a coagulometer (Start4, Diagnostica Stago) according to the protocols of the manufacturer. Briefly, PT was determine by adding 100 µL of Neoplastin (Roche) to 50 µL of plasma; to determine aPTT, 50 µL of plasma were mixed with 50 µL of aPTT reagent (Roche) in the presence of CaCl_2_.

### Platelet aggregation assays

The aggregation of platelets in whole blood collected in the presence of PPACK (40 µg/mL) was performed measuring the changes in impedance (Ohms) produced by physiological agonists in a Chrono-Log instrument. Samples were incubated at 37°C under constant stirring and, after 5 minutes, 10 µM ADP or 5 µg/mL collagen (Chrono-Log) were added. The analysis of data was performed 8 minutes after addition of agonists. The results are means ±SEM of seven different observations in each group.

Collagen-induced aggregation of isolated platelets was measured in a final volume of 800 µL of PRP diluted to 1∶2 with PBS buffer.

### Platelet adhesion and spreading assays

To determine the adherence of platelets to immobilized ligands 100 µL of 5×10^8^ platelets/mL were added to 96-well microtiter flat-bottomed plates coated with fibrinogen (5 µg/mL) and incubated at 37°C for 30 minutes. Non-adherent platelets were removed by aspiration and wells were washed 3 times with 200 µL of PBS buffer.

To study the platelet spreading 100 µL of a platelet suspension containing 5×10^7^/mL platelets was added to microtiter plates coated with fibrinogen (5 µg/mL) and allowed to adhere for 1 hour at 37°C. Non adherent cells were removed by washes with PBS buffer. The adhered platelets were fixed with 3.7% PFA, permeabilized with 0.2% Triton X-100 in PBS buffer, washed twice with PBS and stained for F-actin with FITC-conjugated phalloidin for 20 minutes and washed at least 3 times with PBS. Fluorescence was visualized using an Olympus IX-50 inverted microscope with a monochromatic light source and images taken with a DP70 digital camera (Olympus). The images were analyzed using the ImageJ software [24].

### *In vitro* perfusion experiment

To analyze the formation of platelet thrombi we perfused whole blood diluted to 1∶2 with Tyrode's buffer containing 1% of serum albumin, through parallel plastic chambers (Ibidi, Germany) coated with fibrinogen. Blood was perfused by aspiration with a syringe pump (Harvard Apparatus) at an estimated shear stress of 5 dyne/cm^2^ (μ-slide III 0.1 Luer) or 20 dyne/cm^2^ (μ-slide I 0.1 Luer). Images were taken with an Olympus digital camera. The quantification of platelets adhered to immobilized substrates was carried out with the ImageJ software [24]. We first generated a list of the total number of particles without setting any limits to size or circularity. The particles were sorted by size. We had previously estimated the size of a platelet to be ∼900 pixels. We, then, sorted the aggregates in the groups according to their sizes: small, between 1000–5000 pixel; intermediates, 5000–10000 pixel; large, 10000–30000, and, finally, very large aggregates, greater than 30000 pixel.

To assess adhesion of platelets onto immobilized collagen under flow conditions, ∼25×10^6^ washed platelets suspended in Tyrode's containing 1 mM CaCl_2_ were labeled with calcein-AM (10 µg/mL) (Molecular Probes, Invitrogen). The labeled platelets were perfused through a parallel flow chamber (μ-slide VI 0.1 Luer) precoated with 100 µg/mL of collagen (Chrono-log) at an estimated shear stress of 20 dyne/cm^2^. Non-adhered platelets were removed by washing the chamber with PBS for 5 minutes. To quantify the number of platelets adhered onto collagen the perfusion channel was washed with 50 µL of DMSO and its fluorescence determined. As a blank we used DMSO passed through a collagen coated cell-free perfusion chamber.

### Studies of thrombogenesis *in vivo*

Mice were anesthetized with isofluorane and the yugular veins exposed. A small sample of blood (≤100 µL), extracted from one of the yugular veins, was dropped into a tube containing EDTA and the number of platelets at zero determined in a hemocytometer analyzer Abacus Junior (Diatron Messtechnik GmbH, Austria). Through the same vein we injected a mixture of collagen/epinephrine (150 ng collagen type I (Chronolog) and 15 ng epinephrine (SIGMA) per g of mice body weight). In preliminary experiments we could observe that this proportionate amount of reagents produced a significant drop in the number of circulating platelets without causing the death of the animal. At the indicated time after the injection, blood withdrawn from the yugular vein not previously injected was processed as indicated above and the number of platelets determined.

### Detection of *podxl* gene deletion and Podxl protein

The correct recombination of the *podxl* gene was verified by PCR analysis of genomic DNA extracted from isolated megakaryocytes. The presence of Podxl protein in megakaryocytes was analyzed by western blot using specific monoclonal antibodies [25].

### Megakaryocyte quantification

Mice were perfused through the heart with PBS followed by 4% PFA in PBS and the femurs were immersed in 4% PFA-PBS overnight. The fixed femurs were decalcified and pictures of whole longitudinal sections were taken under light microscope at 20× using a DP70 digital camera mounted on as inverted microscope (IX50, Olympus). The area of megakaryocytes was calculated using the ImageJ software and the cells classified in 5 different groups according to their size. A total of two hundred megakaryocytes from either control or Podxl-null mice were analyzed.

### *In vitro* differentiation of megakaryocytes

Bone marrow cells were flushed from femurs and tibias with Tyrode's buffer, dissociated and cultured in IMDM with 5% fetal bovine serum, 50 ng/mL TPO, 2 mM L-glutamine, 50 U/mL penicillin, and 50 µg/mL streptomycin. Megakaryocytes were recovered by passing the suspension through a discontinuous density gradient of albumin.

### Megakaryocytes ploidy

TPO-differentiated megakaryocytes were labeled with a FITC-conjugated anti-CD41 antibody. The cells were fixed in 70% ice-cold ethanol and stained with propidium iodide solution (50 µg/mL) in the presence of RNAse A (100 µg/mL). The ploidy distribution was determined by 2-color flow cytometry (FACS EPICS XL).

### Megakaryocyte adhesion and proplatelet formation

 TPO-differentiated megakaryocytes were seeded onto collagen-coated coverslips (100 µg/mL), allowed to adhere for 3 hours at 37°C, and fixed in 2% PFA for 15 minutes. The cells were incubated with blocking buffer (0.2% BSA, 0.02% saponin in PBS). Stress fibers were labeled by incubating the cells with phalloidin-AF488 (1/40).

 The percentage of megakaryocytes forming proplatelets was determined by differential interference contrast (DIC) microscopy using a Leica inverted microscope (objective 40×/0.55; Leica Microsystems). Several photographs of the cell were taken and analyzed. Megakaryocytes were examined from 3 independent cultures.

## Results

**Figure 2:**
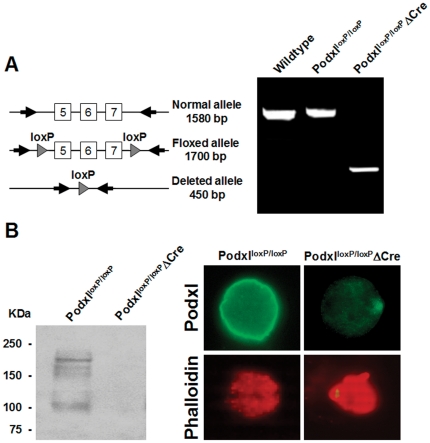
Deletion of the *podocalyxin* gene in megakaryocytes. (A) PCR amplification of genomic DNA from either control or Podxl-null megakaryocytes. (B) In left panel, western blot of megakaryocyte lysates. In upper right panels, immunofluorescence of control and Podxl-null megakaryocytes labeled with anti-Podxl. In lower panels, megakaryocytes from normal of Podxl-null mice stained for F-actin with labeled phalloidin. Fluorescence was visualized using an Olympus IX-50 inverted microscope with a monochromatic light source and images taken with a DP70 digital camera (Olympus).

The ablation of the *podxl* gene in megakaryocytes resulted in no apparent phenotype and the rates of growth and fertility were normal. To assess the presence of the *podxl* gene, we amplified genomic DNA from megakaryocytes of either control (*podxl* ^lox/lox^:: Control) or Podxl-null mice (*podxl* ^lox/lox^:: Pf4-Cre). Floxed allele yielded a fragment of 1,700 bp whereas the deleted allele generated a fragment of 450 bp (Fig. 2A). By western blot we detected a main band of ∼165 KDa in lysates from control megakaryocytes, presumably native Podxl, and other immunoreactive bands of smaller size. In contrast, no immunoreactive signals were detected in lysates from Podxl-null mice (Fig. 2B, left panel). The absence of immunoreactive bands suggests that the smaller bands detected in the control could be partially glycosylated Podxl. Podxl-null megakaryocytes showed no reactivity against anti-Podxl, whereas the control cells showed a strong staining all over the plasma membrane (Fig. 2B, right panel). The pattern of phalloidin staining indicates a similar distribution of the cytoskeleton in both conditions.

Hemograms showed no statistical differences between the controls and Podxl-null mice (results not shown).

### Number and function of megakaryocytes

**Figure 3:**
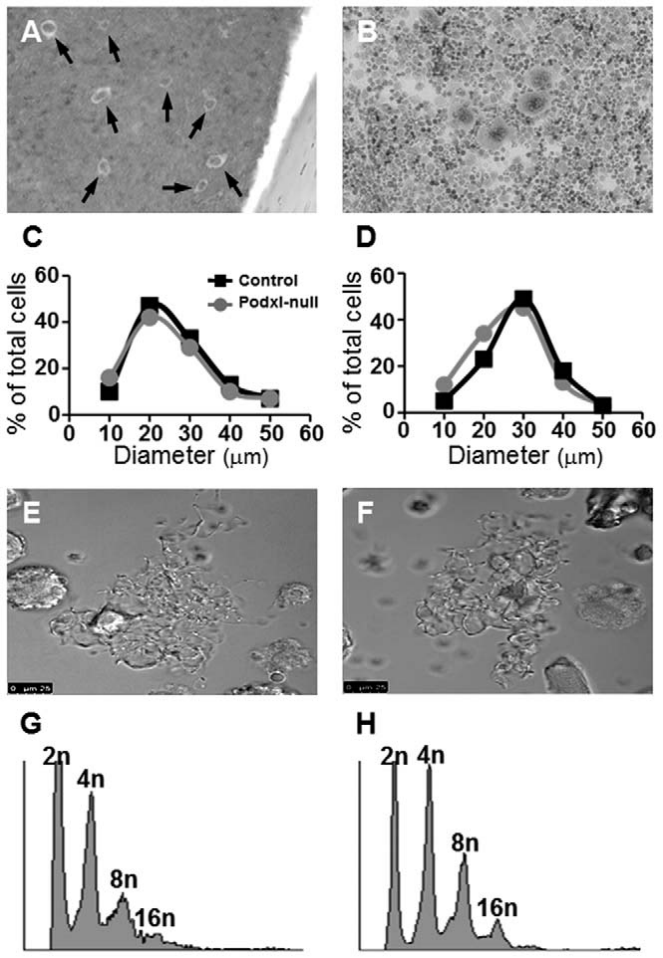
Size and DNA distribution of megakaryocytes *in situ* and differentiated *in vitro*. Megakaryocytes from a decalcified femur (A) and from a bone marrow culture (B). Because the number of megakaryocytes was similar in control and Podxl- null mice, for the sake of clarity, only images from the controls are represented. Size distribution of megakaryocytes *in situ* (C) or in culture (D) determined by wide-field microscopy. Formation of proplatelets in control (E) or in Podxl-null mice (F). DNA distribution in control (G) or Podxl-null megakaryocytes (H).

 To determine the number and shape of megakaryocytes, longitudinal sections of decalcified femurs were stained with Giemsa and the number and areas of megakaryocytes from several sections were analyzed using the ImageJ software. *In vitro* differentiated megakaryocytes were stained with Giemsa and analyzed as described in Methods. The number of megakaryocytes per bone marrow surface area in the Podxl-null mice did not differ from the control. To assess the maturation of megakaryocytes we distributed the megakaryocytes in groups according to sizes ranging from 10 to 60 µm. The number of megakaryocytes in control and podxl-null mice showed no significant differences, neither in decalcified bone marrow sections nor in megakaryocytes differentiated *in vitro* (Figs. 3A,B). Differences in the size-distribution of megakaryocytes in cultured cells or in bone marrow most likely are the result of different states of maturation in each case (Figs. 3C,D).

The adherence of cultured megakaryocytes onto immobilized fibrinogen was similar in controls and Podxl-null megakaryocytes. Transformation of megakaryocytes into proplatelets was observed in both cases (Figs. 3E,F). In agreement with these data, the polyploidy of Podxl-null cultured megakaryocytes did not differ from the control (Figs. 3G,H).

### Bleeding and experimental thrombosis

In view that Podxl seems to enhance the cellular motility and cellular interactions [16], [26] we studied whether the loss of Podxl would alter the platelet function. We found that bleeding time in Podxl-null mice increased by 87% over the control values, and that the carotid closure time induced by ferric chloride was almost double in Podxl-null than in control mice (Table 1). Moreover, the acute systemic thrombosis induced by (i.v.) injection of a mixture of collagen and phenylephrine produced a significantly smaller reduction in the number of circulating platelets in Podxl-null mice than in the controls (Table 2).

**Table 1:** Bleeding and carotid closure times.

	Bleeding time (s)	Carotid closure time (s)
**Control**	40±25(12)	441±25(8)
**Podxl-null**	263±81(8)	922±186(8)
**% increment**	87%	109%
	p<0.05	p<0.01

Bleeding time was measured in animals anesthetized by isofluorane inhalation. A small fragment of the tail (approx. 5 mm) was excised and the tail immersed in isotonic buffer at 37°C, recording the time until bleeding stopped. The carotid closure time was determined by placing a small piece of paper (∼1 mm^2^) soaked with 2% FeCl_3_ onto the artery for two minutes and the blood flow was continuously monitored. A closure time was defined as the time needed for the blood flow be stopped at least for three minutes. The number of observations is shown in parentheses.

**Table 2:** Fall in circulating platelets induced by the i.v. injection of collagen and phenylephrine.

Time (min)	0	2′	% Fall in blood platelets
**Control**	909.4±90.7	398.3±50.3	56.5±2.9
**Podxl-null**	825.6±63	433.1±24.9	46.8±3.6*

Blood was collected from the yugular vein of isofluorane-anesthetized mice followed by i.v. injection of 150 ng collagen type I plus 15 ng of epinephrine per g of mice body weight, as described in Methods. At the indicated time blood was withdrawn from the contralateral yugular vein and the number of platelets determined. Seven animals were analyzed in each group. The values are means ±SEM. By t-test * p<0.05.

**Figure 4:**
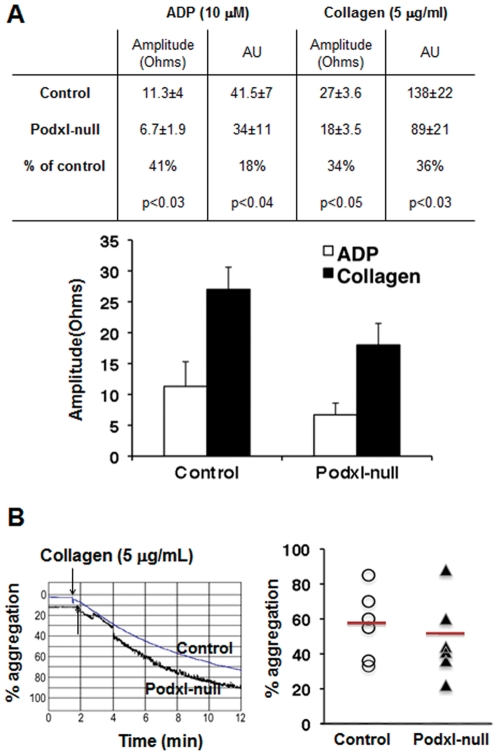
Aggregation of control and Podxl-null platelets. (A) Whole blood-platelet aggregation. Platelet aggregation in whole blood withdrawn in the presence of PPACK (40 µg/mL) was performed by measuring the changes in impedance produced by 10 µM ADP or 5 µg/mL collagen, using a Chrono-Log instrument. Amplitude was the impedance aggregation at 8 minutes; AU, area under curve. The results are means +/−SEM of seven different observations in each group. (B) Collagen-induced aggregation of isolated platelets. Representative aggregation traces from control and Podxl-null platelets treated with collagen (5 µg/mL) as described in Methods. Arrows indicate the addition of the agonist. The graph represents the aggregation values and means from 6 mice of each genotype. No statistical differences were observed.

The platelet content of αIIbβ3, GPIb-V-IX, GPVI and integrin alpha2 chain (CD49b) receptors in Podxl-null mice was similar to that of controls (Table 3) but the aggregation of Podxl-null platelets, analyzed in PPACK-uncoagulated whole blood, showed diminished (30–40%) responses to either ADP or collagen (Fig. 4A). However, no significant differences were detected between control and Podxl-null mice in the collagen-induced aggregation of isolated platelets (Fig. 4B).

**Table 3:** Platelet content of surface receptors.

	αIIbβ3	GPIbα	GPVI	GPIa
**Control**	50.8±7	9.1±1.5	3.14±0.05	5.35±0.43
**Podxl-null**	49.6±6	9.0±1.2	3.03±0.07	5.70±0.52

Platelets rich plasma was incubated with anti-GPIbα-DyLight649 antibody (Xia.G5), anti-αIIbβ3-FITC (Leo.F2), FITC-labeled rat anti-mouse GPVI and FITC-labeled rat anti-mouse integrin alpha2 chain (CD49b) or control isotype-matched mouse IgGs for 30 minutes at room temperature (Emfret Analytics, Eibelstadt, Germany). The samples were then washed, fixed with 1% PFA, and analyzed in a cytometer EPICS XL FACS. The results are means ±SEM of 4 different observations in duplicate. Not significant differences were detected between control and Podxl-null platelets.

**Figure 5:**
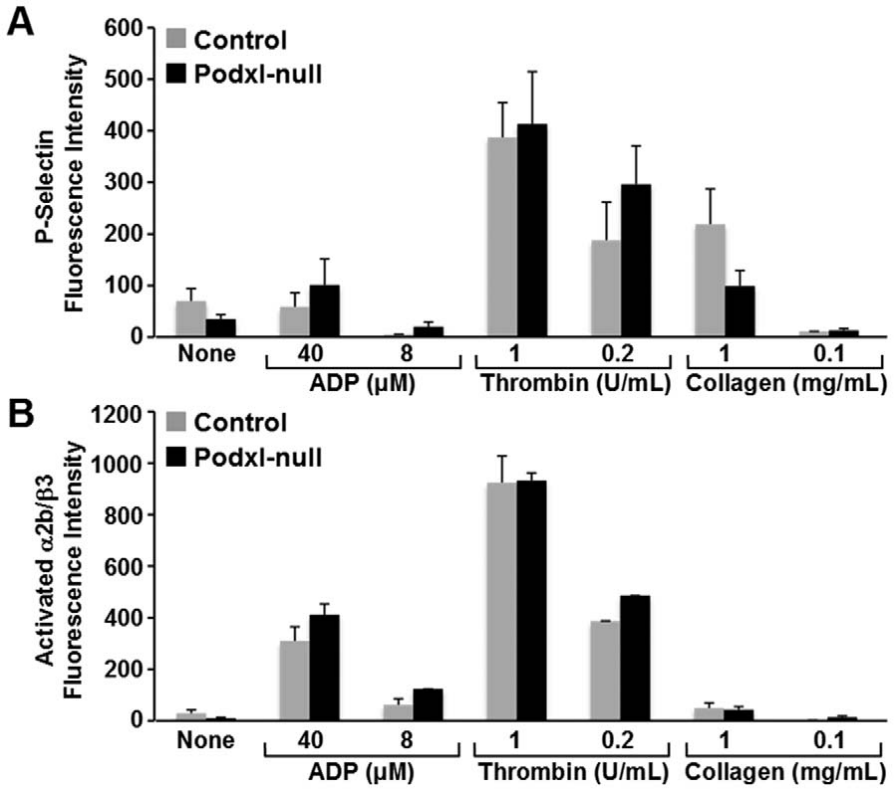
Effect of agonists on the surface exposure of P-selectin or binding of activation dependent antibody JON/A. To determine the platelet responsiveness to agonists we used washed platelets incubated with ADP, collagen or thrombin at the indicated concentrations. Surface exposure of P-Selectin (A) and state of activation of αIIbβ3 (binding of antibody JON/A) (B) were analyzed by flow cytometry. At least 4 experiments were performed and the values are means ±SEM.

The agonist-induced activation of washed platelets, assessed by measuring the binding of the activation-sensitive antibody JON/A or the surface exposure of P-selectin, was similar in Podxl-null and control platelets (Fig. 5). Moreover, the surface exposure of phosphatidylserine, as indicated by the binding of annexin V-FITC after collagen (5 µg/mL) or thrombin (1 U/mL) activation, was also similar in Podxl-null and control platelets (results not shown).

### Platelet adhesion under static conditions and under flow

**Figure 6:**
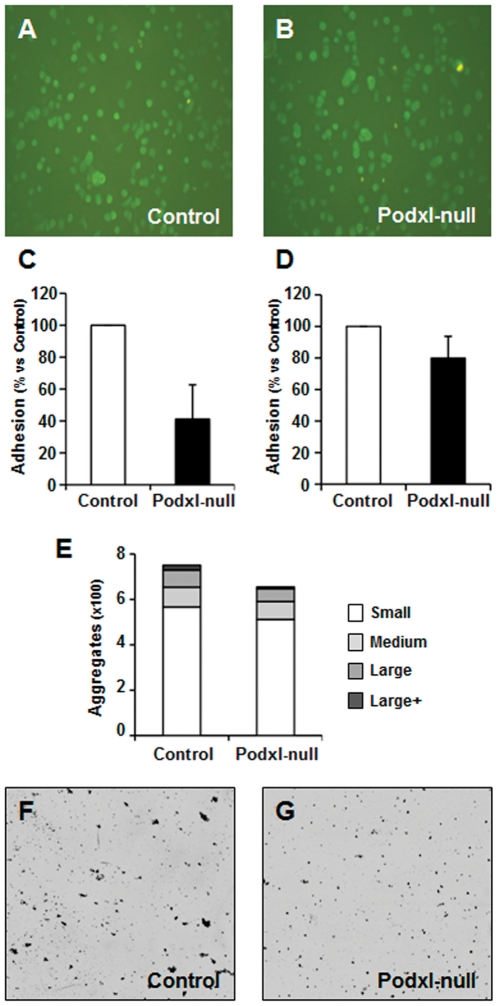
Adherence of platelets to immobilized fibrinogen under parallel flow perfusion conditions. Platelets from control (A) and Podxl-null mice (B) were allowed to adhere onto fibrinogen-coated plates for 30 minutes, fixed and labeled with anti-αIIbβ3. For adhesion under flow, blood was diluted 1∶2 with Tyrode's buffer and perfused by aspiration through a parallel plastic chamber coated with fibrinogen at an estimated shear stress of 5 dyne/cm^2^ (C,F,G) or 20 dyne/cm^2^ (D,E). The adhesion of platelets and formation of aggregates was quantified by the analysis of images using the software ImageJ (http://rsb.info.nih.gov/ij/) as described in Methods. Representative images of platelet adhesion under shear stress of 5 dyne/cm^2^ in control (F) and Podxl-null mice (G).

Figs. 6A,B show that static adhesion stimulated by 10 µM ADP was similar in control and Podxl-null washed platelets, and that in both cases platelets showed morphological features of activation.

For adhesion experiments under flow, citrate-uncoagulated blood diluted with Tyrode's buffer was perfused in plastic parallel flow chambers coated with fibrinogen at an estimated shear stress of 5 dyne/cm^2^ (Figs. 6C,F,G) or 20 dyne/cm^2^ (Figs. 6D,E). The plates perfused with Podxl-null mice blood showed a statistical significant decrease in the number of aggregates.

**Figure 7:**
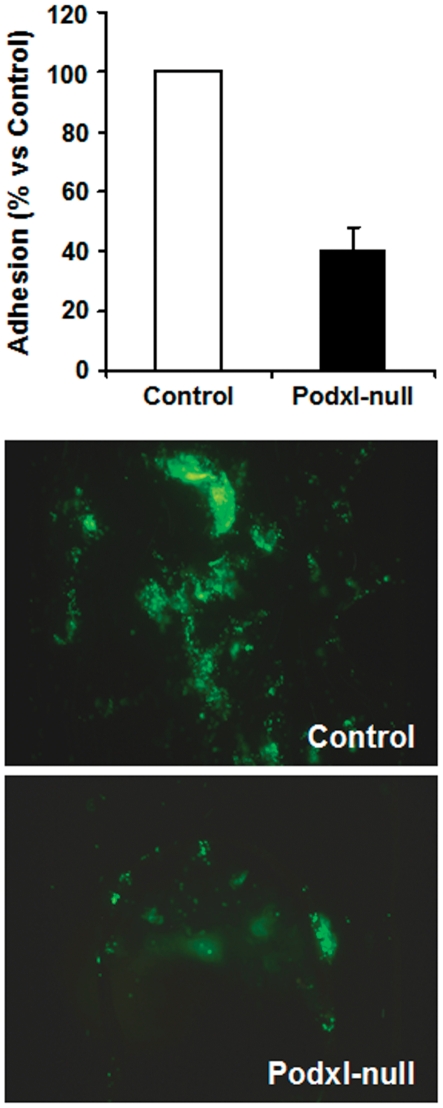
Adherence of platelets to immobilized collagen perfused through a parallel flow chamber. Control or Podxl-null platelets were labeled with calcein-AM, diluted with Tyrode's buffer, and perfused by aspiration through a parallel plastic chamber coated with collagen at an estimated shear stress of 20 dyne/cm^2^. The adhesion of platelets and formation of aggregates was quantified either by the analysis of images using the software ImageJ or by washing the perfusion chamber with DMSO and measuring the fluorescence intensity, as described in Methods. The lower panels show representative images of control and Podxl-null platelet aggregates.

Similar results were obtained when calcein-labeled platelets were perfused under high shear stress (≥20 dyne/cm^2^) in a parallel flow chamber coated with collagen. In five different experiments, the adhesion of Podxl-null platelets to collagen was approximately 40% of the control values (Fig. 7).

## Discussion

 Podocalyxin (Podxl) is a heavily sialylated and sulfated plasma membrane sialoprotein. To investigate the functional role of Podxl in platelets we have generated mice with a megakaryocyte-restricted deletion of the *podxl* gene. The ablation of the *podxl* gene was verified by PCR amplification of genomic DNA from megakaryocytes differentiated *in vitro*. The efficiency of Pf4-Cre in the recombination of floxed genes was further verified by measuring Podxl protein in megakaryocyte lysates. The platelet Podxl-null mice showed normal rates of growth and fertility with no apparent phenotypical traits. The blood cell counts, number of bone marrow megakaryocytes, polyploidy and proplatelet formation did not show statistical differences between the Podxl-null mice and the controls. Despite this apparent normality, the Podxl-null mice showed prolonged bleeding time, reduced platelet aggregation in response to ADP or collagen, retardation of the ferric chloride-induced closure of the carotid artery and a significant decreased in the thrombogenic response *in vivo* induced by (i.v.) injection of collagen and phenylephrine. Moreover, adherence and formation of aggregates onto fibrinogen or collagen-coated plates in a whole blood perfusion system was reduced in Podxl-null platelets compared to the controls. According to these observations, the removal of Podxl from megakaryocytes altered the control of hemostasis. To note, overexpression of Podxl in megakaryocytes and platelets [27] has been recently reported to produce just the opposite effects, this is, decreased bleeding time and increased agonist-induced platelet aggregation. Thus, these observations reinforce the idea that Podxl may contribute to the control of hemostasia.

Whatever the mechanism of Podxl action, one should expect a decreased aggregation of platelets to be caused either by defective or deficient platelet receptors. However, the platelet content of fibrinogen receptor (αIIbβ3) and von Willebrand factor receptor (GPIb-V-IX) in Podxl-null platelets was similar to the controls. Moreover, the agonist-induced activation of washed platelets, as indicated by surface exposure of P-selectin, binding of the activation-dependent moAb JON/A to αIIbβ3, or membrane exposure of phophatidylserine, did not show differences between control and Podxl-null platelets.

 Since the number of platelet receptors and their ability to respond to agonists *in vitro* were normal in the Podxl-null mice, then, what is causing a decreased efficiency of the hemostatic control in the absence of Podxl?. At least two simultaneous processes are involved in controlling the hemostasia: firstly, the formation of platelet clots; secondly, the formation of fibrin clots by the plasma coagulation sequence. A role for Podxl in fibrin clot formation appears to be excluded in view that Podxl-null mice showed similar plasma values of PT and aPTT than the control group.

The adhesion and aggregation of platelets and the thrombogenic occlusion of vessels can take place in mice lacking vWF and fibrinogen [28]. Thus, factors other than interaction of the main ligands, vWF and fibrinogen, with their respective platelet receptors must be involved in the formation of a platelet clot. Moreover, GPIbα contributes to arterial thrombosis by mechanisms independent of its main ligand, vWF [29]. The extracellular protein fibronectin was postulated to be one of these factors, since reduced plasma fibronectin produced defective thrombus formation [30]. On the other hand, both plasma and platelet proteins seem to contribute to vWF/fibrinogen-independent platelet aggregation [31]. More than 20 cytoplasmic proteins have been reported to interact with the carboxyterminal (CT) domains of either αIIb or β3 glycoproteins [32]. Plasma membrane or transmembrane proteins like CD40L, Gas6, semaphorin 4D (CD100) or Platelet Endothelial Aggregation Receptor 1 (PEAR1), have been reported to be involved in the control of thrombus formation and/or stabilization. These antecedents highlight the redundancy of the system controlling the hemostasia and give support to the possibility that Podxl was one more of the factors controlling the hemostasia acting either directly or through interactions with other membrane proteins. It should also be considered that Podxl could influence the coagulation through interactions with glycoproteins of the coagulation cascade.

 Podxl is heavily sialylated and changes in sialic acid content of platelets have been reported to alter platelet function [33], [34]. Even if the loss of platelet Podxl would not change significantly the total content of sialic acid, the possibility should be considered that the absence of specific protein interactions could alter the intracellular platelet signaling. Moreover, the actions of Podxl in enhancing adhesion and motility were abolished in mutated cells in which the *O*-sialylation was impeded [16].

 Podxl could also act in controlling αIIbβ3 signaling pathway through cytoplasmic interactions. The CT domain of Podxl is linked to the cytoskeleton through the scaffold protein EBP50/NHE-RF (ezrin-binding protein/Na^+^H^+^ exchange regulatory factor) [18], [35] and colocalizes with vinculin [16]. On the other hand, talin, a cytosolic protein with a FERM domain is specifically involved in the activation and linking of integrin αIIbβ3 to the actin cytoskeleton [35]–[37]. Talin binds vinculin with high affinity [37]–[40]. Vinculin and talin are two pivotal components of the focal adhesions that may indirectly activate integrins. Both, Podxl and αIIbβ3, colocalize with vinculin at the leading edges of the cell and at regions of intercellular contacts [16]. Thus, it is tempting to speculate that a decrease functional efficiency of αIIbβ3 in Podxl-null mice could arise from a functional imbalance of FERM and/or scaffold proteins.

 Stable transfection of human Podxl in CHO cells enhances cell adhesion, motility and cellular interactions in a selectin and integrin-dependent manner, suggesting that Podxl could act as a co-activator of platelet αIIbβ3 [16]. On high endothelial venules, Podxl is involved in lymphocyte homing through L-selectin interactions [18]. Since the Podxl effects on cell adhesion are selectin-dependent, the possibility should also be considered that Podxl-selectin interactions would be a significant factor in the control of hemostasis.

 To conclude, mice with null-Podxl megakaryocytes generated by the Cre/LoxP gene targeting methodology showed perturbed hemostatic reponses: increased bleeding time, diminished aggregation of platelets in response to agonists, retarded ferric chloride-induced closure of the carotid artery, decreased systemic thrombosis and decreased adhesion and formation of platelet aggregates under parallel flow conditions. Since Podxl-null platelets showed normal receptors content and normal activation by agonists *in vitro*, the perturbation of hemostasis in the absence of Podxl could be the result of a decreased efficiency of platelet adhesiveness produced by the loss of sialic acid and/or selectin-dependent interactions.
